# Evaluation of the Efficacy and Safety of Satralizumab in a Pregnant NMOSD Patient With AQP4/MOG‐IgG Dual Seropositive: A Case Report

**DOI:** 10.1002/acn3.70275

**Published:** 2025-12-05

**Authors:** Yeting Luo, Shuhua Xie, Xianghong Liu

**Affiliations:** ^1^ Department of Neurology, Ganzhou People's Hospital Nanchang University Nanchang Jiangxi China

**Keywords:** AQP4‐IgG, dual positivity, MOG‐IgG, NMOSD, parturition, satralizumab

## Abstract

Neuromyelitis Optica Spectrum Disorder (NMOSD) is a chronic autoimmune neuroinflammatory disease, typically characterized by antibodies against aquaporin 4 (AQP4‐IgG) or myelin oligodendrocyte glycoprotein (MOG‐IgG). Simultaneous seropositivity for both antibodies in a single patient is exceedingly rare. We present a dual AQP4‐IgG/MOG‐IgG seropositivity case who was treated with satralizumab throughout the whole preconception‐to‐postpartum course, to evaluate the effectiveness and safety of satralizumab, especially during the perinatal period. A 34 year‐old female, initially presenting with decreased visual acuity in the left eye, was diagnosed with NMOSD as both AQP4‐IgG and MOG‐IgG seropositive. With traditional treatment of corticosteroids and mycophenolate mofetil, her vision gradually recovered and overall condition stabilized. Due to the desire for conception, her treatment regimen was transitioned to satralizumab monotherapy. Three months later with five doses of satralizumab, she successfully conceived and delivered a healthy female infant at 38 weeks' gestation. Satralizumab treatment was continued throughout the preconception‐to‐postpartum course. All routine and perinatal assessments were within normal limits, and 4 months postpartum, the condition of both mother and child remained stable, further supporting the favorable effectiveness and safety of satralizumab in this case. The coexistence of AQP4‐IgG and MOG‐IgG in an NMOSD patient represents an extremely rare and complex clinical scenario. When fertility is desired, the selection of disease‐modifying therapy must carefully balance effectiveness and safety. In such cases, satralizumab may serve as a viable option, supported by promising real‐world data.

## 1 Introduction

Neuromyelitis Optica Spectrum Disorder (NMOSD) is a rare autoimmune demyelinating disease of the central nervous system, classically presenting as optic neuritis (ON), transverse myelitis or brainstem syndromes. Patients typically experience relapsing courses and accumulated irreversible disability. Most cases are dominated by the presence of antiaquaporin 4 immunoglobulin G (AQP4‐IgG), accounting for 60%–80% of NMOSD patients. Among AQP4‐IgG negative individuals, 21%–27% identified antimyelin oligodendrocyte glycoprotein immunoglobulin G (MOG‐IgG), which is also called MOG antibody‐associated disease (MOGAD) [1]. Concomitant detection of both antibodies is exceptionally rare, only accounting for approximately 0.15% of all cases [2]. The clinical phenotypes and long‐term outcomes of these dual seropositive cases remain poorly characterized and require further study. In addition, NMOSD predominantly affects women of childbearing age; pregnancy and parturition are associated with an increased relapse rate, especially during the first 3 months postpartum [3, 4]. Thus, when conception is desired, therapeutic strategies must carefully balance effectiveness, maternal safety, and fetal risk. Robust evidence indicates that elevated interleukin‐6 (IL‐6) plays a significant role in the pathophysiology of NMOSD. Pharmacologic blockade of the IL‐6 signaling cascade can effectively prevent relapses [5]. Satralizumab is a novel anti‐IL‐6 receptor monoclonal recycling antibody, administered subcutaneously, approved for the treatment of AQP4‐IgG positive NMOSD [6]. A case has been reported that satralizumab had favorable effectiveness and safety in a pregnant female NMOSD patient and no evidence indicated satralizumab transfer to umbilical cord blood, infant serum or breast milk [7]. However, dual‐antibody positivity, pregnancy and parturition constitute an exceptionally rare clinical scenario. To further confirm the effectiveness, maternal safety and fetal risk of satralizumab in this setting, we report a case with concomitant AQP4‐IgG and MOG‐IgG who received continuous satralizumab monotherapy throughout the preconception‐to‐postpartum course with favorable effectiveness and safety. This case provides valuable insights into the clinical management of such rare presentations.

## 2 Case Report

**FIGURE 1:**
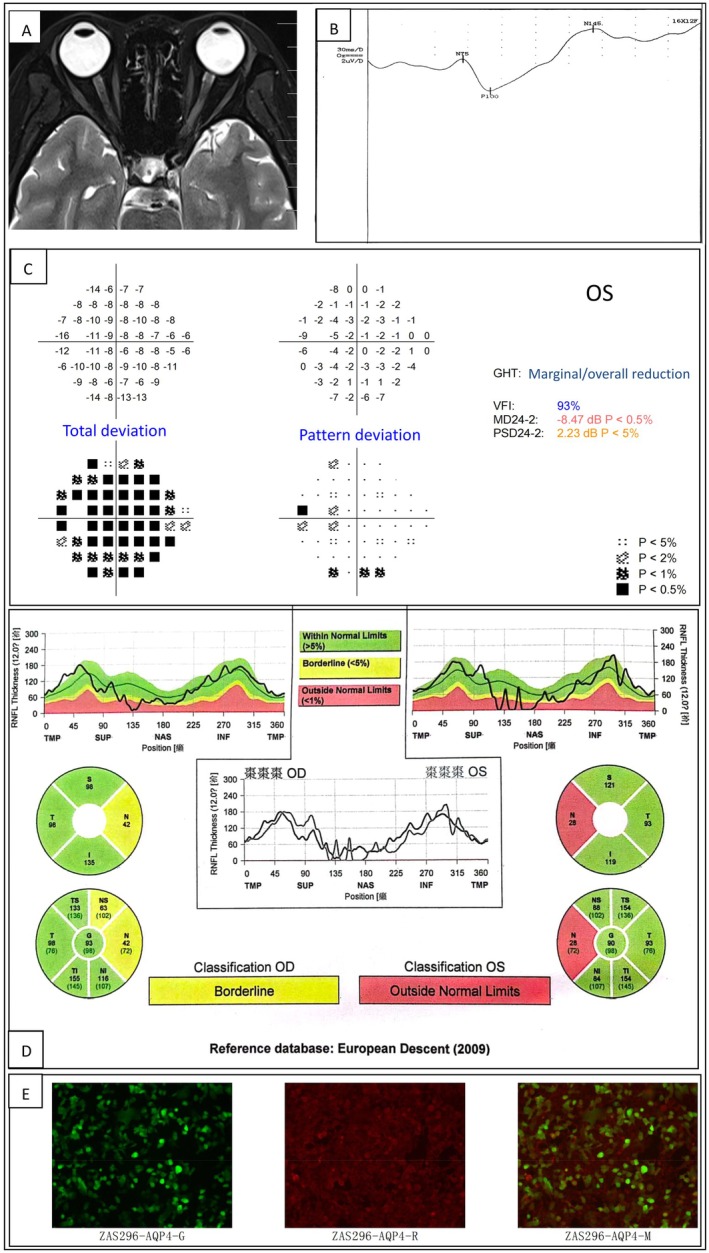
Presentation of selected clinical findings of the patient. (A) Magnetic resonance imaging (MRI) of the patient's optic nerve revealed no abnormal findings. (B) Visual evoked potential (VEP) testing demonstrated prolonged latency of the left‐sided VEP100 waveform, which is consistent with impaired conduction along the left visual pathway. (C) The visual field examination of the left eye demonstrated the presence of visual field defects across all quadrants. (D) The retinal nerve fiber layer (RNFL) assessment revealed bilateral thinning, with more pronounced involvement of the left eye. (E) Laboratory testing confirmed the presence of serum aquaporin‐4 immunoglobulin G (AQP4‐IgG) antibodies (cell‐based assay, titer 1:32).

The patient was a 34 year‐old female with a history of Hashimoto's thyroiditis and bilateral high myopia. She initially experienced left eye pain upon ocular movement on May 6, 2023, followed by progressive visual acuity decline. By May 9, her visual impairment had reached its peak severity, with only light perception remaining. On May 8, she presented to the ophthalmology department of our hospital with the aforementioned symptoms. Upon admission, the direct pupillary light reflex of the left eye was diminished, whereas the indirect light reflex was preserved. Laboratory testing revealed the presence of serum AQP4‐IgG (fixed cell‐based assay, titer 1:32) and absence of serum MOG‐IgG (fixed cell‐based assay). Routine and biochemical analyses of the cerebrospinal fluid yielded normal results. The cerebrospinal fluid interleukin‐6 concentration was 2.00 pg/mL, which falls within the normal reference range (reference value: < 7.00 pg/mL). Oligoclonal bands are present in both serum and cerebrospinal fluid, with identical banding patterns observed in both compartments. Cerebrospinal fluid testing for AQP4‐IgG and MOG‐IgG has not yet been performed. Magnetic resonance imaging (MRI) of the orbits, brain, and spinal cord, performed both pre‐ and postcontrast administration, revealed no abnormal findings. Visual evoked potential (VEP) testing demonstrated prolonged latency of the left‐sided VEP100 waveform, consistent with impaired conduction along the left visual pathway. Visual field examination of the left eye revealed visual field defects across all quadrants, varying in severity. Retinal nerve fiber layer (RNFL) assessment showed bilateral thinning, with more pronounced involvement of the left eye. Some of the results are shown in (Figure 1).

The patient was diagnosed with NMOSD due to the positive AQP4‐IgG. Intravenous methylprednisolone was administered (500 mg daily for 7 days, then 250 mg daily for 3 days, followed by 125 mg daily for 3 days), but visual acuity improved only from light perception to finger‐counting at 30 cm, with a corrected vision of 0.02. The patient was subsequently referred to the neurology department for plasma exchange therapy, which was administered over five sessions. Following the treatment, corrected visual acuity improved to 0.4. Maintenance therapy was initiated with a combination of mycophenolate mofetil and oral prednisone (10 mg once daily), resulting in 12 months free from relapse and no evidence showing disease recurrence.

**FIGURE 2:**
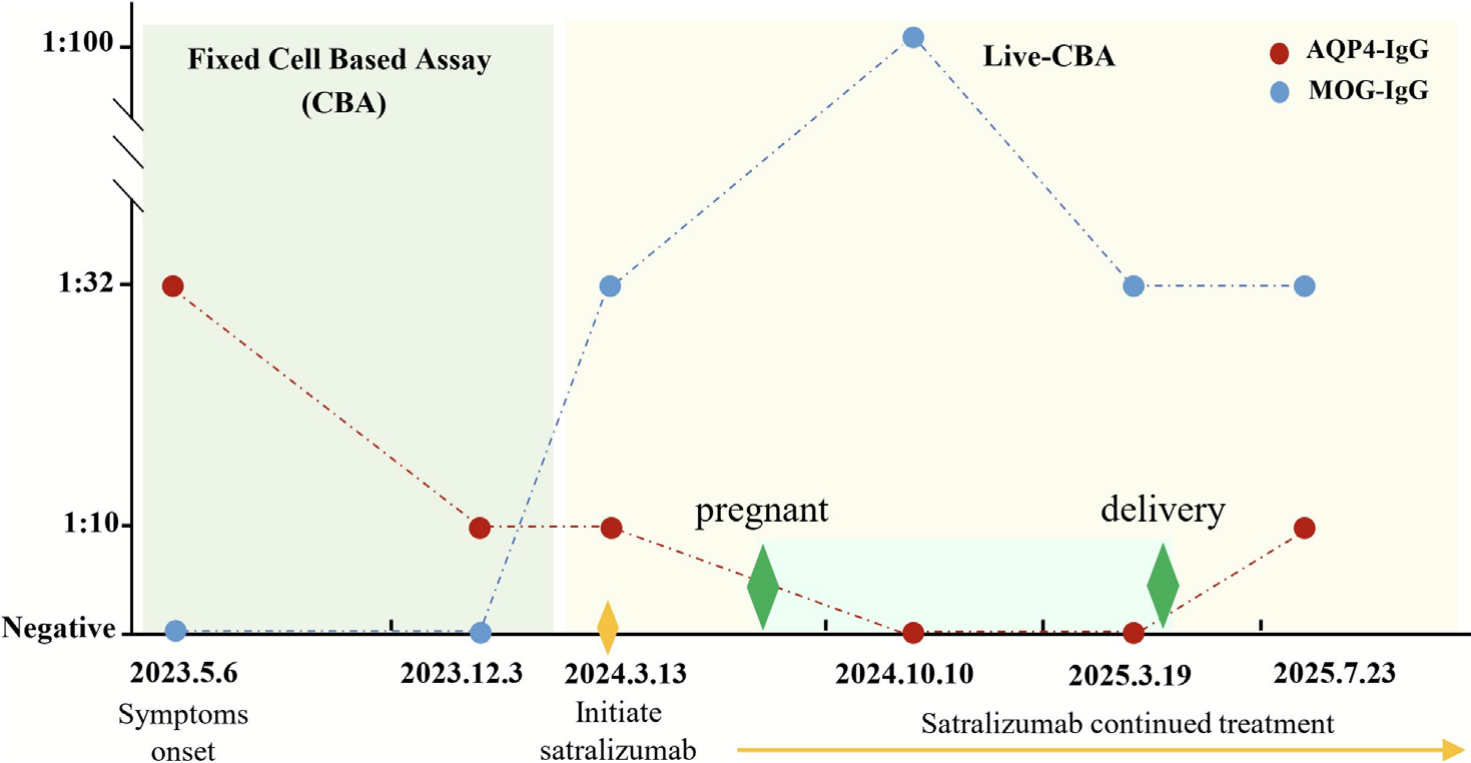
Illustration of the titer variation of two antibodies in the patient.

On March 13, 2024 the patient attended a preconception consultation. Compared with the fixed‐cell assay, the live‐cell assay demonstrates higher sensitivity for AQP4‐IgG detection and greater clinical relevance for MOG‐IgG detection [8, 9]. Therefore, the live‐cell assay has been implemented for both AQP4‐IgG and MOG‐IgG testing in this case and all subsequent evaluations. In AQP4‐IgG testing, live cells expressing full‐length, conformationally intact recombinant human AQP4 protein are used as antigen substrates, with transfected cells serving as negative controls. For MOG‐IgG detection, an IgG Fc‐specific secondary antibody is employed to ensure specific signal detection. Laboratory testing revealed that the serum AQP4‐IgG titer had decreased to 1:10 (live cell assay, reference value is negative). Notably, serum MOG‐IgG, which was previously negative when assessed by fixed cell‐based assay now tested positive with a titer of 1:32 using the live cell assay (reference value is negative). Given the pathogenic role of interleukin‐6 (IL‐6) in both AQP4‐IgG and MOG‐IgG disorders, mycophenolate mofetil was discontinued and initiated with satralizumab. Three months later, with five doses of satralizumab, the patient successfully conceived. Seven months after initiation of satralizumab treatment, a routine follow‐up assessment on October 10, 2024 revealed that the patient had become seronegative for serum AQP4‐IgG, while serum MOG‐IgG remained positive at a titer of 1:100. On March 19, 2025, the routine follow‐up test results of the patient showed that the serum AQP4‐IgG was negative and the serum MOG‐IgG titer was 1:32. Satralizumab continued throughout gestation without any symptoms recurrence and no adverse events were observed; all the routine prenatal tests were normal. On April 7, 2025, she delivered a healthy female infant at 38 weeks' gestation, weighing 2450 g, Apgar score of 10. A total of 14 doses of satralizumab were administered throughout the entire fertility course. On July 23, 2025, 4 months postpartum, the follow‐up evaluation revealed the serum AQP4‐IgG titer of 1:10 and the serum MOG‐IgG titer of 1:32. Currently, it has been 5 months postpartum; both mother and child are in good condition and the patient's visual function is well‐preserved with no clinical relapses occurred. The titer variation of two antibodies is shown in (Figure 2).

## 3 Discussion

AQP4‐IgG is the principal driver of NMOSD pathology, triggering complement‐dependent cytotoxicity that injures astrocytes and precipitates downstream demyelination [10, 11]. Nevertheless, a proportion of patients with NMOSD phenotypes remain seronegative for AQP4‐IgG. Within this group, MOG‐IgG identifies a distinct entity whose pathophysiology resembles type II demyelinating lesions seen in multiple sclerosis (MS), mediated primarily by T cells and macrophages‐driven inflammation [12]. Although the pathogenesis differs from AQP4‐IgG‐mediated disease, MOG‐IgG‐associated optic neuritis can follow a relapsing course reminiscent of NMOSD. Nonetheless, it more frequently manifests as bilateral optic nerve involvement, severe papilledema, MRI findings of more extensive optic nerve enhancement, and optic disc edema that can be pronounced (occurring in up to 86%) and even peripapillary hemorrhage [13, 14, 15, 16, 17, 18, 19, 20], and it tends to be more common in children and young adults and is typically associated with relatively favorable clinical outcomes [12]. Notably, several studies have reported cases of coexistence of AQP4‐IgG and MOG‐IgG. In addition to the optic neuritis, these dual‐positive patients may develop area postrema syndrome and longitudinally extensive transverse myelitis [21, 22]. A South Asian referral‐center study of 1935 NMOSD patients identified only three cases (0.15%) with both antibodies [2]. All three patients presented with severe clinical phenotypes, including area postrema syndrome, bilateral optic neuritis, and transverse myelitis, suggesting that the clinical manifestations in dual‐positive patients are more consistent with classical Neuromyelitis Optica (NMO) rather than MOGAD [2]. In the present case, the patient manifested solely with optic neuritis. Intriguingly, the initial fixed cell‐based assay detected AQP4‐IgG while yielding a negative MOG‐IgG result, whereas the subsequent live cell assay demonstrated dual positivity for both antibodies, underscoring the latter's superior sensitivity. Consequently, all subsequent monitoring was conducted using the live‐cell assay. The results showed that AQP4‐IgG remained negative throughout pregnancy but became positive again postpartum, while a stable MOG‐IgG titer of 1:32 was observed. Considering the relapse‐free course and persistently positive MOG‐IgG, we further believed that AQP4‐IgG was the primary pathogenic driver in this case, whereas MOG‐IgG served as ancillary. However, the possibility that MOG‐IgG could become dominant in the future cannot be excluded; therefore, the treatment must be designed to address both AQP4‐IgG and MOG‐IgG‐mediated diseases simultaneously.

Emerging evidence indicates that glucocorticoids have limited therapeutic effectiveness in patients who are dual‐positive for AQP4 and MOG antibodies [2]. Among conventional interventions, plasma exchange has shown potential benefits in this subgroup [2]. In recent years, monoclonal antibody biologics (mAb) targeting B cells, complement, or the IL‐6 pathway have been increasingly utilized in AQP4‐IgG seropositive patients with great effectiveness and safety, yet robust data in dual‐positive individuals remain scarce. Rituximab (RTX), an anti‐CD20 mAb, is widely used as a first‐line therapy and has demonstrated efficacy in published dual‐positive cases [2], but its utility is tempered by infusion‐related and hypersensitivity reactions [23]. Obinutuzumab (OFA), a novel fully humanized anti‐CD20 mAb, has been successfully employed in a small case series of dual‐positive patients with marked clinical improvement and no significant adverse events [24], suggesting that it may be a valuable alternative for those intolerant to RTX. More recently, eculizumab (C5 inhibitor) and satralizumab (IL‐6‐receptor blocker) have shown favorable efficacy in NMOSD [25, 26], while their role in MOGAD remains fully elucidated. Because their targets are not antibody‐restricted [27], both agents could plausibly benefit dual‐positive patients, although prospective data are lacking. Satralizumab is developing an indication for MOGAD (NCT05271409). In the present case, due to the upregulation of IL‐6 and its downstream inflammatory cascade in both AQP4‐IgG and MOG‐IgG‐related disorders, the treatment strategy of satralizumab was justified. Our report provided additional real‐world evidence supporting the clinical efficacy of satralizumab in this patient population.

NMOSD predominantly affects women of childbearing age, so the fetal safety of any maintenance therapy is paramount. A previous retrospective study demonstrated that, among patients with neuroimmune disorders exposed to anti‐CD20 agents during pregnancy, the preterm birth rate was 45%, the incidence of congenital abnormalities was 3.3%, and the risk of neonatal B‐cell depletion was elevated; however, the correlation with anti‐CD20 agents remains uncertain [28]. In contrast, preclinical studies in pregnant monkeys have shown that the administration of satralizumab, at both high and low doses, does not significantly prolong gestation, increase miscarriage rates, or elevate stillbirth rates [29]. In addition, the safety of satralizumab during pregnancy is further supported by clinical case reports. Two case reports have documented full‐term deliveries without apparent neonatal complications in patients with continuous satralizumab treatment throughout gestation [7, 30]. One case specifically measured drug levels and confirmed satralizumab concentration in umbilical cord blood was below the assay's lower limit of quantification (0.200 μg/mL), and during exclusive breastfeeding, the infant's serum drug concentration remained undetectable [7]. Our case further supports the maternal and fetal safety of satralizumab during the whole preconception‐to‐postpartum course.

## 4 Conclusion

This case report describes a rare instance of AQP4‐IgG and MOG‐IgG dual seropositive NMOSD in a patient treated with satralizumab throughout gestation, resulting in stable maternal disease and uncomplicated fetal development. The patient's clinical course highlights several key observations: First, dual positivity for AQP4‐IgG and MOG‐IgG remains an exceptionally rare phenotype. In this case, the patient presented with severe optic neuritis, consistent with reports of aggressive clinical manifestations in dual‐seropositive individuals. Second, satralizumab monotherapy effectively maintained disease stability during pregnancy, with no relapses observed. This suggests that satralizumab may exert disease‐modifying effects in the context of dual antibody positivity, most likely through IL‐6 receptor blockade—a mechanism that targets a shared inflammatory pathway in both AQP4‐IgG– and MOG‐IgG–mediated pathogenesis. Third, the safety profile of satralizumab in pregnancy is further supported in this case. Fetal growth and development were unremarkable, with no evidence of drug‐related adverse events in the neonate. This is consistent with prior animal studies and limited clinical reports demonstrating minimal transplacental transfer and absence of neonatal harm, reinforcing satralizumab as a potential therapeutic option for pregnant NMOSD patients requiring maintenance therapy.

## Author Contributions

Liu X made substantial contributions to the conception, design, and critical revision of the study. Luo Y was responsible for the acquisition of clinical data and drafting of the manuscript. Xie S contributed to the analysis and interpretation of the data. All authors read and approved the final manuscript.

## Funding

The authors have nothing to report.

## Ethics Statement

The study protocol was approved by the Ethics Committee of Ganzhou People's Hospital. Written informed consent was obtained from the patient for publication of the patient's history and associated images.

## Consent

Authors give consent for publication.

## Conflicts of Interest

The authors declare no conflicts of interest.

## Data Availability

The data that support the findings of this study are available from the corresponding author upon reasonable request.
